# Pregnancy outcomes and perinatal complications of Asian mothers with juvenile idiopathic arthritis – a case-control registry study

**DOI:** 10.1186/s12969-020-0404-8

**Published:** 2020-01-23

**Authors:** Shang Jun Zhang-Jian, Huang-Yu Yang, Meng-Jun Chiu, I-Jun Chou, Chang-Fu Kuo, Jing-Long Huang, Kuo-Wei Yeh, Chao-Yi Wu

**Affiliations:** 1 Chang Gung University, College of Medicine, Taoyuan, Taiwan; 2 Department of Nephrology, Chang Gung Memorial Hospital, Taoyuan, Taiwan; 3 Center for Artificial Intelligence in Medicine, Chang Gung Memorial Hospital, Taoyuan, Taiwan; 4 Division of Neurology, Department of Pediatrics, Chang Gung Memorial Hospital, Taoyuan, Taiwan; 5 Department of Rheumatology, Chang Gung Memorial Hospital, Taoyuan, Taiwan; 6 Division of Allergy, Asthma, and Rheumatology, Department of Pediatrics, Chang Gung Memorial Hospital, Taoyuan, Taiwan

**Keywords:** Juvenile idiopathic arthritis, Outcomes research, Pregnancy

## Abstract

**Backgrounds:**

In order to provide juvenile idiopathic arthritis (JIA) patients with better pre-conceptional and prenatal counselling, we investigated the obstetrical and neonatal outcomes among women with Asian descent.

**Methods:**

Through the linkage of Taiwan National Health Insurance database and National Birth Registry, we established a population-based birth cohort in Taiwan between 2004 and 2014. In a case control study design, first children born to mothers with JIA are identified and matched with 5 non-JIA controls by maternal age and birth year. Conditional logistic regression was used to calculate odds ratios for maternal and neonatal outcomes crude and with adjustment.

**Results:**

Of the 2,100,143 newborn, 778 (0.037%) were born to JIA mothers. Among them, 549 first-born children were included in this research. Our result suggested that babies born to mothers with JIA were more likely to have low birth body weight, with an adjusted OR of 1.35(95% CI: 1.02 to 1.79) when compared to babies born to mothers without. No differences were observed in other perinatal complications between women with and without JIA including stillbirth, prematurity, or small for gestational age. The rate of adverse obstetrical outcomes such as caesarean delivery, preeclampsia, gestational diabetes, postpartum hemorrhage and mortality were also similar between the two.

**Conclusions:**

Adverse obstetrical and neonatal outcomes were limited among Asian mothers with JIA. Intensive care may not be necessary for JIA mothers and their newborns.

## Background

Juvenile idiopathic arthritis (JIA) is the most prevalent debilitating rheumatic disease of childhood [1]. It comprises heterogeneous subtypes of diseases with complex immunopathology that leads to joint inflammation before the age of 16 [2]. The prevalence of JIA worldwide ranges from 16 to 150 /100,000 with female predominance [3–5]. According to the Taiwan National Health Insurance (NHI) database, the overall prevalence rate of JIA is 29.7 to 33.8/100,000. Approximately 27.1/100,000 women suffered from JIA in Taiwan during the period of 1999 to 2010 [6, 7].

Increasing attentions have been raised on reproduction related issues in patients with rheumatic diseases [8, 9]. Despite the success of conventional and new biological treatments, a substantial percentage of JIA patients will have ongoing active disease into adulthood. Prolong inflammation can lead to placenta insufficiency and resulted in intrauterine growth restriction, miscarriage, preterm birth, pre-eclampsia and small for gestational age (SGA) [8, 10, 11]. Additionally, the use of disease-modifying anti-rheumatic drugs and biologics for disease control also raised the concern for pregnancy interruption [9, 12]. Clear understanding of the potential adverse events not only provide patients with better pre-conceptional and prenatal counselling, but likely ease JIA mothers from inexplicable worries.

Studies investigating the risk of JIA related pregnancy morbidities have been reported [13–16]. However, only very few discussed about the potential neonatal complications. Moreover, all of the available studies at present are based on the Caucasian population. Considering the differences of genetic background can significantly influence the distribution of JIA subtypes and long-term disease outcome, previous conclusions may not be directly applied to those with Asian descent [17–21].

Aimed to provide mothers suffered from JIA with a better pre-conceptional and prenatal counselling, we took advantage of the NHI database and National Birth Registry in Taiwan. We examined the pregnancy outcomes as well as perinatal complications of neonate born to mothers with JIA in a population-based cohort incorporating patients with Asian origin exclusively.

## Material and method

This study was approved of by the Institutional Review Board of the Chang Gung Memorial Hospital (201800358B0) and the data holders of the NHI database and the National Birth Registry in Taiwan. Patient consent was exempted since all study subjects were de-identified and completely anonymoused.

### Data source

The primary data sources were the NHI database and the National Birth Registry. With the help from the NHI administration, we linked the two databases by using the national identification number which is unique to each resident of Taiwan. Since the NHI database contain registration and claims data of the entire population, respective data for the mothers and their children were extracted. The linkage of mother-child relationships was ascertained using the National Birth Registry. The traceable data were encrypted by the data holder to prevent confidentiality break.

The Taiwan NHI database was established in 1996, and contains the health data on approximately 99.5% of all Taiwanese population [22]. The health data recorded in the NHI database includes gender, date of birth, family relationships, dates of medical visits, clinical diagnosis, medical prescriptions, examinations, procedures and operations, as well as medical expenditures and expenses. The NHI has a special program for maternity care including antenatal health examinations, pregnancy outcomes and postpartum care.

The National Birth Registry records information of live births and stillbirths of neonate above 500 g of weight or elder than 20 weeks in gestation. It contains data of infants’ gender, birth order, birth body weight and height, Apgar scores, gestational age and neonatal abnormalities. The neonatal outcome and maternal outcome documented in the National Birth Registry have been proven to be a valid source for researchers [23].

### Study population

In Taiwan, JIA was diagnosed according to the International League of Associations for Rheumatology (ILAR) classification criteria by the pediatric rheumatologists [24]. Mothers with JIA were identified with the following International Classification of Disease version 9 (ICD-9-CM) codes: rheumatoid arthritis (ICD-9: 714.0), rheumatoid arthritis with systemic involvement (ICD-9: 714.2), juvenile chronic polyarthritis (ICD-9: 714.3), ankylosing spondylitis (AS) (ICD-9: 720.0), psoriatic arthropathy (PsA) (ICD-9: 696.0) or inflammatory bowel disease (IBD) associated arthritis (ICD-9: 713.1) with a concurrent IBD code (ICD-9: 555 or 556). Because no single ICD-9-CM code was designed for enthesitis related arthritis (ERA), it is commonly accepted by physicians in Taiwan to code ERA with AS (ICD-9: 720.0) only, AS and RA (ICD-9: 714.0), AS and juvenile chronic polyarthritis (ICD-9: 714.3) or IBD associated arthritis (ICD-9: 713.1) in cases with IBD. With such coding, ERA was found to account for 39% of all JIA cases [6]. This is similar to the percentage of ERA previously reported in Taiwan [24, 25]. Moreover, rheumatoid arthritis with systemic involvement (ICD-9: 714.2) was used by physicians in Taiwan for cases diagnosed with systemic JIA. To improve classification accuracy, two or more records of pediatric rheumatologist diagnosis within 6 months before the age of 16 were required.

Pregnancies among JIA patients were identified and the cases with gaps between two deliveries less than 6 months or longer than 20 years were excluded from another. A total number of 2,100,143 pregnancies and 1,468,318 mothers were identified during the period of 2004 to 2014. The total pregnancy population is divided into two groups: women with JIA and those without. A total of 778 babies were born to 549 mothers with JIA. To avoid possible confounding factors and for case control selection, only the first child from either group was included for further analysis. In addition, considering maternal age as a potential confounding factor and to bypass the possible bias resulted from case selection, we matched 5 non-JIA cases as controls to one JIA cases by maternal age and birth year for further investigation [26].

### Study outcomes

Neonatal and maternal outcomes were both examined. Neonatal outcomes include low birth weight (< 2500 g), SGA(<10th percentile for the same gestational age), large for gestational age (LGA) (>90th percentile for the same gestational age), apgar score at 1 and 5 min, prematurity (< 37 weeks), stillbirth, fetal distress and fetal abnormalities including central nervous system malformations and chromosome abnormalities. Small and large for gestational age were calculated according to the nomogram summarizing data collected from all livebirths in the National Birth Registry between 2004 and 2014.

Maternal outcomes include death (death within 30 days post-partum), pregnancy-related hypertension, antepartum hemorrhage, severe postpartum hemorrhage, chorioamnionitis, caesarean delivery, preeclampsia, and gestational diabetes. Maternal deaths were verified by utilizing the National Death Registry in Taiwan. It kept records on the causes of death for all deceased citizens. The accuracy of the coding has previously been validated [27, 28].

### Covariate definitions

Maternal age, country of origin, place of residence, income level, occupation, obstetric history, and Charlson comorbidity index were included as maternal covariates. The place of residence was based on the urbanization of 369 towns and districts in Taiwan, which was divided into 3 categories: urban, suburban, or rural. Income levels were estimated by the business income for employers and the payrolls for the employees. It was categorized into 5 quintiles of which the first quintile being the highest paid. Occupations were classified into 5 categories: (1) dependents (2) civil servants, teachers and military personnel/veterans; (3) professionals and non-manual workers; (4) manual workers; and (5) others. Charlson comorbidity index is a summary measure scored on a number of medical conditions including diabetes, chronic renal diseases and cardiovascular diseases..etc., with an integer weighted score from one to six [29]. Substituting the use of individual comorbidity variables by the summation of the weighted comorbidity scores have been widely accepted by researchers for comorbidity adjustment in outcome studies utilizing administrative health data [30]. Charlson comorbidity index was based on the validated version for ICD-9 codes and the rheumatological disease category is excluded from the Charlson comorbidity index in this study [31].

### Data analysis

The rate of each outcome was compared between groups of mothers with and without JIA using a conditional logistic regression to estimate the odds ratio (OR) and the corresponding 95% confidence intervals (CI). A model adjusted for potential confounders including: infant sex, residence, income, occupation and Charlson comorbidity index was performed. Two-sided test with 5% level of significance was used for all statistical hypotheses. All analyses were performed using SAS v. 9.4 (SAS institute, Cary, NC).

## Result

### Baseline population

**Fig. 1:**
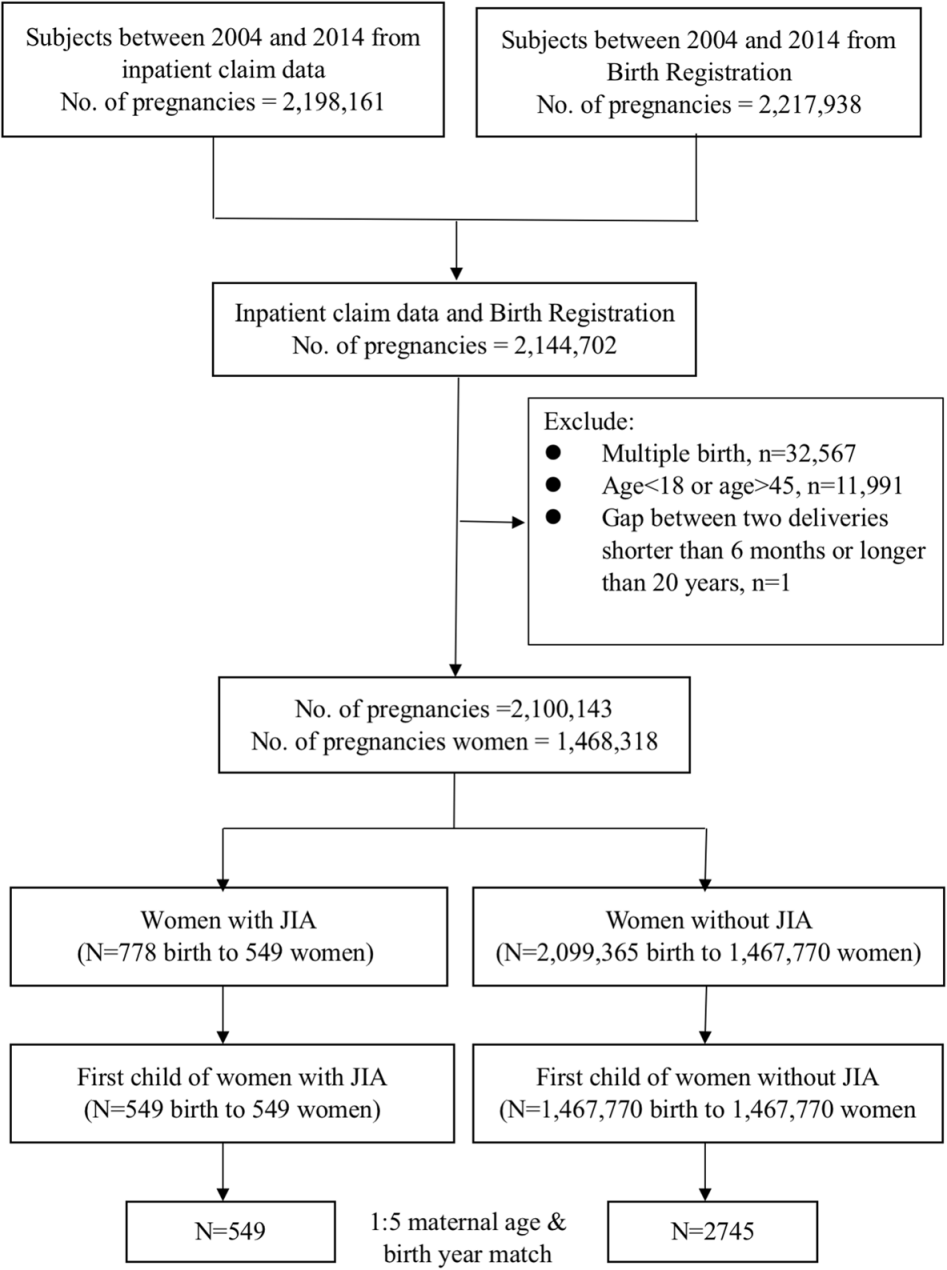
Flow Chart

In our study, a total of 2,100,143 babies were born to 1,468,318 mothers between 2004 and 2014. Among them, 778 babies were delivered from 549 mothers who met our JIA diagnostic criteria (Fig. 1). The baseline population and characteristics of the 549 and 2745 matched pregnancies among women with or without JIA were demonstrated in Table 1. The average maternal age of delivery is 24.4 ± 3.4 years old and more babies were delivered by the JIA mothers in the later years. There were no significant differences between the two groups except for the Charlson comorbidity index (*P* < 0.05) (Table 1). Women with JIA were more likely to have comorbidities compared to those without. Two out of 549 JIA mothers received biologics during pregnancy.

**Table 1:** Baseline characteristics of pregnancies among women with or without juvenile idiopathic arthritis

	With JIA (*n* = 549)		Without JIA (*n* = 2745)		*p* value
Age at pregnancy, mean (SD), years	24.4	(3.4)	24.4	(3.4)	1.00
< 25	298	(54.3)	1490	(54.3)	
25–34	251	(45.7)	1255	(45.7)	
Birth year, No. (%)					1.00
2004	14	(2.6)	70	(2.6)	
2005	19	(3.5)	95	(3.5)	
2006	25	(4.6)	125	(4.6)	
2007	21	(3.8)	105	(3.8)	
2008	28	(5.1)	140	(5.1)	
2009	46	(8.4)	230	(8.4)	
2010	35	(6.4)	175	(6.4)	
2011	52	(9.5)	260	(9.5)	
2012	84	(15.3)	420	(15.3)	
2013	86	(15.7)	430	(15.7)	
2014	139	(25.3)	695	(25.3)	
Male infant	285	(51.9)	1396	(50.9)	0.65
Foreign nationals, No. (%)	0	0	340	(12.4)	
Place of residence, No. (%)					0.15
Urban	319	(58.1)	1536	(56.0)	
Suburban	175	(31.9)	980	(35.7)	
Rural	55	(10.0)	229	(8.3)	
Income levels, No. (%)					0.79
Quintile 1	128	(23.3)	691	(25.2)	
Quintile 2	140	(25.5)	726	(26.5)	
Quintile 3	138	(25.1)	664	(24.2)	
Quintile 4	94	(17.1)	445	(16.2)	
Quintile 5	49	(8.9)	219	(8.0)	
Occupation, No. (%)					0.14
Dependents	182	(33.2)	1011	(36.8)	
Civil servants, teachers, military personnel, and veterans	7	(1.3)	21	(0.8)	
Non-manual workers and professionals	192	(35.0)	962	(35.1)	
Manual workers	97	(17.7)	387	(14.1)	
Other	71	(12.9)	364	(13.3)	
Charlson comorbidity index	0.07	(0.4)	0.03	(0.2)	< 0.05*
0	525	(95.6)	2698	(98.3)	
1	19	(3.5)	40	(1.5)	
≥ 2	5	(0.9)	7	(0.3)	

*Abbreviation*: *JIA* juvenile idiopathic arthritis, *SD* standard deviation

* indicated *p*-value < 0.05

### Neonatal outcome

According to the World Health Organization definition, infants born to mothers with JIA were more likely to be low in birth weight (< 2500 g) as compared to those born to mothers without (OR = 1.44; 95% CI =1.09 to 1.90). As shown in Table 2, after adjusting for infant sex, Charlson comorbidity index, urbanization, income, occupation and maternal nationality, an increased risk of low birth weight remain to be found among those born to mothers with JIA (aOR = 1.35; 95% CI =1.02 to 1.79). However, babies born to JIA mother were found to weight 25.3 g less than those reference population without significance (*P* = 0.25). Furthermore, no difference was found in other neonatal outcomes, including still birth, prematurity, small or large for gestational age, apgar score < 7 at 1 and 5 min, fetal distress and fetal abnormalities.

**Table 2:** Comparison of fetal-neonatal outcomes between pregnancies of women with and without juvenile idiopathic arthritis

	No. of events (%)				Crude OR (95% CI)		Adjusted OR (95% CI)^a^	
	With JIA (n = 549)		Without JIA (n = 2745)					
Stillbirth	4	(0.7)	23	(0.8)	0.87	(0.30–2.51)	0.92	(0.32–2.69)
Low birth weight (< 2500 g)	64	(11.7)	222	(8.1)	1.44	(1.09–1.90)	1.35	(1.02–1.79)*
Prematurity (< 37 week)	49	(8.9)	197	(7.2)	1.24	(0.91–1.70)	1.19	(0.87–1.64)
Small for gestational age	86	(15.7)	353	(12.9)	1.22	(0.96–1.54)	1.16	(0.91–1.47)
Large for gestational age	29	(5.3)	203	(7.4)	0.71	(0.48–1.05)	0.72	(0.48–1.07)
Apgar score 1 min (< 7)	9	(1.6)	66	(2.4)	0.68	(0.34–1.37)	0.66	(0.32–1.33)
Apgar score 5 min (< 7)	1	(0.2)	14	(0.5)	0.36	(0.05–2.71)	0.25	(0.03–2.08)
Fetal distress	32	(5.8)	170	(6.2)	0.94	(0.65–1.37)	0.96	(0.65–1.41)
Fetal abnormalities, any	38	(6.9)	201	(7.3)	0.95	(0.67–1.34)	0.91	(0.64–1.29)

*Abbreviation*: *N/A* not applicable, *JIA* juvenile idiopathic arthritis, *OR* odds ratio, *CI* confidence interval

^a^Adjusted for infant sex, Charlson comorbidity index, urbanization, income, occupation, maternal nationality

* indicated *p*-value < 0.05

### Maternal outcome

Maternal outcomes between women with and without JIA were shown in Table 3. The rate for caesarean delivery was 28.2% in women with JIA and 26.6% in the control population. No differences were observed before and after adjustment (OR = 1.06; 95%CI = 0.89 to 1.26 and aOR = 1.07; 95%CI = 0.89 to 1.27). Other pregnancy and delivery related complications such as preeclampsia, gestational diabetes or severe postpartum hemorrhage show no statistically significant difference between the two groups (Table 3).

**Table 3:** Comparison of maternal outcomes between pregnancies of women with and without juvenile idiopathic arthritis

	No. of events (%)				Crude OR (95% CI)		Adjusted OR (95% CI)^a^	
	With JIA (n = 549)		Without JIA (n = 2745)					
Preeclampsia	17	(3.1)	54	(2.0)	1.57	(0.91–2.71)	1.42	(0.82–2.46)
Gestational diabetes	24	(4.4)	145	(5.3)	0.83	(0.54–1.27)	0.75	(0.48–1.16)
Pregnancy-related hypertension	24	(4.4)	87	(3.2)	1.38	(0.88–2.17)	1.24	(0.79–1.96)
Antepartum hemorrhage	55	(10.0)	247	(9.0)	1.11	(0.83–1.49)	1.09	(0.81–1.47)
Severe postpartum hemorrhage	25	(4.6)	99	(3.6)	1.26	(0.81–1.96)	1.20	(0.77–1.87)
Chorioamnionitis	5	(0.9)	17	(0.6)	1.47	(0.54–3.99)	1.34	(0.49–3.66)
Caesarean delivery	155	(28.2)	731	(26.6)	1.06	(0.89–1.26)	1.07	(0.89–1.27)
Death ≤30 days post-partum	0	(0.0)	0	(0.0)		N/A		N/A

*Abbreviation*: *N/A* not applicable, *JIA* juvenile idiopathic arthritis, *OR* odds ratio, *CI* confidence interval

^a^Adjusted for infant sex, Charlson comorbidity index, urbanization, income, occupation, maternal nationality

## Discussion

To our knowledge, this is the first study to investigate JIA related pregnancy outcomes and perinatal complications focus on women with Asian origin. Using a nationwide population-based cohort of which the data were extracted from the Taiwan NHI database and the National Birth Registry, we recruited 549 JIA mother-and-neonate pairs accommodating single ethnicity. As compared to previous study analyzing less than 10 Asian cases, the relatively large sample size in the present study provided sufficient statistical power to detect differences in the risk of adverse birth outcomes and pregnancy morbidities comparing women with and without JIA [26].

Physical and psycho-social stress resulted from chronic disease and the fear of neonatal and pregnancy related complications can potentially influence the willingness of women to give birth to children. In fact, a decrease in pregnancy rate has been reported by *Wallenius* et al. among women with JIA [32]. Although our data showed that mothers with JIA were more likely to give birth to neonate with low birth weight (< 2500 g), babies born to JIA mother were found to weight only 25.3 g less than those reference population. Compared to the average neonatal birth weight which is around 3130 g in Taiwan, the difference is minimal [33]. Furthermore, because no difference was found in other neonatal outcomes, including still birth, prematurity, fetal distress and fetal abnormalities … etc., we concluded that adverse obstetrical and neonatal outcomes were fairly limited among Asian mothers with JIA if any. Our findings disburdened JIA mothers and clinical physicians through the pregnancy process.

Unlike previous studies which have shown an increased risk of caesarean section (CS), pre-eclampsia, postpartum hemorrhage, preterm labor, miscarriages and abortion in JIA mothers, none of the analyzed adverse obstetrical outcomes were notable in the present study [13–16].. Furthermore, neonates born to mothers with JIA had previously been discovered with an increased risk for prematurity, SGA and congenital neural tube defect [14, 15, 26, 34]. Studies by *Chen* and *Remaeus,* however, failed to observe significant effects of maternal JIA on the risk of SGA, low Apgar score or severe neonatal morbidity [14, 15]. These inconsistencies in the pregnancy and neonatal outcomes may likely resulted from the differences in patients’ genetic background, definition of each morbidity, selection of patients, number of cases and methodological differences in study design. Among all, ethnicity is perhaps an important factor which may significantly influence perinatal outcomes [26]. While pauciarticular arthritis is the most common JIA subtypes among Caucasians, enthesitis-related arthritis is much common among Asians [17–20]. In addition, lower prevalence rate of JIA in Asia has been reported as compared to other parts of the globe [4, 5]. Considering the diverse nature of JIA among patients with different genetic background, *Ringold* et al. analyze the Childhood Arthritis and Rheumatology Research Alliance Registry and found a more favorable outcome among Asians suffering JIA as compared to those white and black children [21]. The discrete disease courses implied a possibility for shorter duration of inflammation and an opportunity for earlier medication withdraw.

According to the previous study, 97.4% of the JIA patients received non-steroidal anti-inflammatory drug (NSAID), 26.7% received intra-articular steroid injection, 73.3% received conventional synthetic disease-modifying antirheumatic drugs (csDMARDs), and 12.8% received biological DMARDs (bDMARDs) during the period of 1995–2010 in Taiwan [24]. Since the introduction of biologics into JIA treatment, issues of infection and concurrent biologic use have become great concern especially in women during pregnancy. However, due to the limited use of biologics before and after *conception and the relative low risk* of serious infections during and after gestation, no study so far have discussed the risk of biologics solely in JIA mothers. Instead, results gather from women with various autoimmune diseases have recently found that the use of biologics during pregnancy is not associated with higher risks of maternal infections but may potentially impact neonatal outcome due to its ability to cross placenta [35, 36]. Further studies, however, is warranted before any final conclusion can be drawn.

JIA patients were under the age of 16 during the period of 2004 to 2014 to meet the diagnostic criteria and be recruited as study subjects in the present study. Therefore, most of the women with JIA were still below 30 years old at time data analysis. This explained why the average maternal age of delivery is lower than the reported age of motherhood in Taiwan [37]. In addition, this also clarified why more babies were being delivered by the JIA mothers as the years progressed. Moreover, the extreme high rate of elective CS in Taiwan possibly masked the significance of CS and instrumental delivery in JIA mothers [38].

Several limitations deserved to be mentioned. First, the database uses the ICD-9 coding system provided by the clinical physicians for diagnosis. Despite a comprehensive inclusion and exclusion criteria, misclassification may still occur. Second, long-term outcomes and treatment responses have been shown to vary between different JIA subtypes [39, 40]. Without detail clinical information and laboratory data, critical factors such as JIA subtype-classification and severity were not included from our analysis. Third, side effects resulted from therapeutic regimen have been shown to significantly contribute pregnancy morbidities in mothers with inflammatory arthritis [41–43]. The statistical power, however, was not enough to analyze the complexity and impact of therapeutic regimen in obstetrical and birth outcomes among JIA mothers with our current cohort. Moreover, because data on maternal smoking habit were not available in the databases, an important confounder was missing for neonatal birth weight adjustment. Finally, the study is based solely on population with Asian ethnicity. Unlike earlier studies that included participants from diverse ethnic groups, limitations existed in the generalizability to extend our observation over cases of different race.

## Conclusions

In conclusion, adverse obstetrical and neonatal outcomes were limited among pregnancies of JIA mothers with Asian origin. JIA mothers and their babies should be advice to follow routine obstetric care without over worrying. Future studies exploring the association between adverse pregnancy outcomes with the subtypes and severity of JIA as well as medications taken during pregnancy are also warranted.

## Data Availability

The datasets generated and/or analysed during the current study are not publicly available due the regulation of the NHI database and the National Birth Registry but are available from the corresponding author on reasonable request.

## Abbreviations

CI: Confidence intervals
CS: Caesarean section
ICD-9: International Classification of Disease version 9
ILAR: International League of Associations for Rheumatology
JIA: Juvenile idiopathic arthritis
LGA: Large for gestational age
NHI: National Health Insurance
OR: Odds ratio
SGA: Small for gestational age
